# Plasma Metabolites and Gut Microbiota Are Associated With T cell Imbalance in BALB/c Model of Eosinophilic Asthma

**DOI:** 10.3389/fphar.2022.819747

**Published:** 2022-05-18

**Authors:** Yumei Zhou, Tieshan Wang, Xiaoshan Zhao, Ji Wang, Qi Wang

**Affiliations:** ^1^ National Institute of TCM Constitution and Preventive Medicine, School of Chinese Medicine, Beijing University of Chinese Medicine, Beijing, China; ^2^ Beijing Research Institute of Chinese Medicine, Beijing University of Chinese Medicine, Beijing, China

**Keywords:** eosinophilic asthma, Tcell imbalance, drug treatment, plasma metabolites, gut microbiota

## Abstract

The pathogenesis of allergic asthma is complex, it is usually caused by immune system imbalance. Th1, Th2, regulatory T cells (Treg) and T helper 17 (Th17) cells have an important role in the pathogenesis of eosinophilic asthma. Yet, the exact role of Th1, Th2, Treg and Th17 cells in eosinophilic asthmatic disease is not fully understood. This study used an untargeted plasma metabolomics combine 16S rDNA technology to identify new biomarkers of plasma metabolites and gut microbiota in ovalbumin-induced eosinophilic allergic asthma in BALB/c mice to further explore the biomarkers in regulating the immune balance or the immune response. We discovered that malate, l-dihydroorotate were associated with Th1/Th2 and Treg/Th17 cells balance, imidazoleacetic acid was associated with Th1/Th2 cell balance, 1,5-anhydro-d-sorbitol was associated with Treg/Th17 cell balance. The results also found that genus *Candidatus Arthromitus of* gut microbiota were associated with Th1/2, Treg/Th17 balance, genus *Ruminiclostridium 6*, they were all associated with Th1/2 and Treg/Th17 cell balance, while the gut microbiota were not associated with penh value which reflect airway hyperresponsiveness (AHR) in the eosinophilic asthma mice model. Interestingly, the plasma metabolite biomarkers of malate, l-dihydroorotate are associated with genus Ruminiclostridium 6, they were all associated with Th1/2 and Treg/Th17 cell balance, while imidazoleacetic acid is associated with genus Ruminiclostridium 6 which is associated with Th1/2 balance. Among the differential plasma metabolites, 1,5-anhydro-d-sorbitol is associated with genus *Ruminiclostridium 6* and genus *Candidatus Arthromitus*. Among them, malate participate in the T cell activation, T cell differentiation and activation may be a new research direction in eosinophilic allergic asthma. We firstly study the gut microbiota and plasma metabolites markers of immune balance in eosinophilic asthma in mice model, laying a foundation for drug treatment in eosinophilic allergic asthma.

## Introduction

More than 300 million people worldwide are suffered from allergic asthma which is a long-term disease. Allergic asthma is also a chronic inflammatory condition characterized by high responsiveness to inhaled allergens in the respiratory tract (Matsumura et al., 2006). The in-depth immunologic characterization of patients and the emergency of biologics agents targeting type2-high cytokines have classified asthma patients into those with a high type 2 inflammatory response (type2-high group) and those with low or no type 2 inflammation (type2-low group). Type 2-high asthma is related to the over-expression cytokines of IL-4, IL-5, IL-9, and IL-13 which are usually produced by the innate immune system which can recognize allergens (including bacteria, viruses and allergens), and are essential in the effect phase of allergic reactions. Type2-high asthma is associated with excessive production of fractional exhaled nitric oxide (FeNO), mucus overproduction, increased eosinophil and mast cell infiltration, bronchial hyperresponsiveness, and excessive synthesis of IgE. In type 2-high allergic asthma, IL-4 and IL-13 are involved in mucus production and goblet cell hyperplasia in lung tissue, airway hyperresponsiveness, eosinophil invasion into the lungs, and IgE over-production (Woodruff et al., 2009; Pavord et al., 2019). The eosinophil in peripheral blood is often used as a marker to the treatment of type 2 asthma in allergic asthma patients. For example, most corticosteroid-treated asthma patients has been found eosinophilia in the sputum (Lambrecht et al., 2019).

There are two main types of allergic asthma, Th2 high asthma is predominantly eosinophilic, while Th2 low is predominantly neutrophilic and paucigranulocytic (Zhang et al., 2022). In allergic asthma patients, the appearance of Th2 cell response, the production of allergen-specific IgE, and the regulation of the recruitment of effector cells to the lung tissue are all related to the continuous immune tolerance of the allergen (Verbsky and Chatila, 2013; Lambrecht and Hammad, 2015). It is also currently believed that Th1/Th2 imbalance is one of the key immunological mechanisms. Increasing genetic and immunological evidence suggests that Treg cells have an essential role in inducing immune tolerance to allergens and preventing the occurrence and development of allergic asthma (Chatila et al., 2000; Torgerson et al., 2007; Jones et al., 2014; Palomares et al., 2014). Treg cells can inhibit allergic inflammation and play an important role in tissue remodeling (Gri et al., 2008; Nonaka et al., 2008). Treg cells can prevent effector T cells from flowing into inflammatory tissues through a cytokine-dependent manner (Ring et al., 2006). Treg can also reduce the induction of Th0/Th1 cells (Trautmann et al., 2002), thus eliminating bronchial epithelial cells and preventing tissue injury. Besides, Treg directly affects B cells through suppress the production of allergen-specific IgE (Meiler et al., 2008a), and inhibit the secretion of TGF-β, IL-10, CTLA-4 or histamine to perform these functions (Meiler et al., 2008b; Sakaguchi et al., 2009). In allergic asthma, it is well accepted that the generation and maintenance of Treg cells, the low expression of suppression cytokines such as IL-10, TGF-β and surface molecules are essential for the pathogenesis and the development of the disease. The induction of allergen tolerance is indispensable for allergic asthma (Palomares et al., 2017).

Th17 cells, as a subpopulation of CD4^+^ T cells which can induce eosinophilic airway inflammation in asthma patients. Many studies have reported that the immune regulation Th17/Treg cells is correlated with asthma severity. Moreover, it has been suggested that Th17 cells could inhibit Treg cell-mediated tolerance and promote airway remodeling. Yet, the exact role of Treg and Th17 cells in eosinophilic asthmatic disease and airway remodeling are not fully understood (Zhao et al., 2013).

Metabolomics can perform high-dimensional molecular atlas analysis of diseases, and it is possible to define the endophenotype of diseases (Swietlik et al., 2021). In this study, we used an untargeted plasma metabolome to identify new biomarkers of plasma metabolites associated with the immune balance of Th1/2 and Treg/Th17 cells in a classical eosinophilic allergic asthma mice model. Besides, we also used 16S rDNA technology to analyze gut microbiota so as to further explore the role of gut microbiota in regulating the immune balance of Th1/2 and Treg/Th17 cells.

The pathogenesis of allergic asthma is complex, we can find OVA, OVA combine aluminum adjuvant, OVA combine Lipopolysaccharide (LPS), HDM (house dust mites) were used to construct allergic asthma mice model, while the immune response in the body are different. Until now, the allergic asthma induced by OVA is mainly predominantly infiltrated by eosinophils which can be demonstrated by my previous study (Zhou et al., 2022), while induced by HDM is maily infiltrated by neutrophils (Ma et al., 2021). When conducting research, different animal models are selected according to the different therapeutic effects of the drug. Although there are too much study focus on the gut microbiota or plasma metabolites in asthma disease, until now we have not found the biomarker of the gut microbiota or plasma metabolites associated with Th1/2 or Treg/Th17 immune balance in the animal study in eosinophilic asthma, which is critical for mechanism research and it is the foundation of drug therapy in eosinophilic asthma disease.

## Methods

### Animals

BALB/c female mice (6–8 weeks old, 18–20 g) were obtained from Beijing Vital River Laboratory Animal Technology Co., Ltd. in China. All the animals were kept and all the animal studies were done according to institutional animal care regulations of Beijing University of Chinese Medicine and conducted according to the AAALAC and the IACUC guidelines.

### Eosinophilic Asthma Mice Model Construction

The Eosinophilic asthma mice model was constructed according to our previously study (Zhou et al., 2022). Briefly, mice were sensitized intraperitoneally with 2 μg ovalbumin (OVA) and 2 mg Imject™ Alum Adjuvant (Invitrogen, Cat#77161) dissolved in 0.2 ml sterile PBS. The control group received 2 mg Alum Adjuvant in 0.2 ml of sterile PBS on 0 and 14 days, respectively. In the challenge phase, mice received 1% OVA dissolved in sterile PBS for 30 min from the 21st to 25th day by aerosol inhalation.

### The Detection of Lung Function, Collection of Bronchoalveolar Lavage Fluid (BALF) and the Serum

The lung function detection was used to evaluate airway hyperresponsiveness (AHR). Briefly, on day 26 post-modeling, AHR was assessed by determining enhanced pause (Penh value). Briefly, mice were placed in the plethysmography chambers of a whole-body plethysmograph (WBP-4MR, TOW, China), after 2, 3 min acclimation, mice were exposed to aerosolized methacholine (Mch) with a series concentrations: 0, 6.25, 12.5, 25, and 50 mg/ml. Non-invasive measurement of airway hyperresponsiveness reflect lung function by whole-body phelthysmography was done according to a previously described approach (Sun et al., 2021).

Twenty-four hours after the last challenge with either OVA or PBS on the 26th day, bronchioalveolar lavage (BAL) of the lung was performed. Samples were centrifugated at 4°C, 1,200 rpm for 5 min resuspended in PBS. The supernatants of BAL fluids were analyzed by multiplex-assay ELISA kits containing IL-4, IL-5, IL-13, IL-17A, IL-6, IL-10, TGF-β and IFN-γ (Luminex, Univ, Cat#T2C0710709). OVA-specific IgE was detected according to the ELISA kit provided by Cayman (Cat#500840). Blood analyzer was used to count the number of Eosinophil cells.

### Histopathology of Lungs

After 4% paraformaldehyde fixation and paraffin embedding of the lung tissue. HE (Hematoxylin and eosin), PAS (periodic acid–Schiff) staining were used to evaluate the inflammation and goblet cells hyperplasia. Inflammation grade was scored according to the following criteria: grade 0 (no inflammatory cells), grade 1 (some inflammatory cells), grade 2 (1-3 layer inflammatory cells surrounded bronchi), grade 3 (4, 5 layer of inflammatory cells surrounded bronchi) and grade 4 (more than five layers of inflammatory cells surrounded most bronchi). The pathological changes were quantified by the percentage of goblet cells in the epithelium using a five-point scoring system: grade 0 (no goblet cells), grade 1 (< 25% of airway), grade 2 (25–50% of airway), grade 3 (51–75% of airway) and grade 4 (>75% of airway). More than five bronchioles were counted in one pathological section, the inflammation scores or goblet cell hyperplasia scores was used in the study (Padrid et al., 1995).

### Detection the Percentage of Th1, Th2, Treg and Th17 Cells

The percentage of Treg and Th17 cells in spleen tissue were detected by flow cytometry. Spleen tissues were prepared into a single cell suspension. CD3 (BD, Cat#557666), CD4 (BD, Cat#552775), CD25 (BD, Cat#558642), IFN-γ (BD, Cat#557735), IL-4 (BD, Cat#562915) were surface staining, while Foxp3 (eBioscience, Cat#17-5773-82) and IL-17A (BD, Cat#564169) were stained after the cell membrane was destroyed by eBioscience Fix/Perm (Cat#00-5523-00) or BD Fix/Perm buffer kit (Cat#554714) respectively. FVS 780 (BD, Cat#565388) was used to identify the live or dead cells. LSR Fortessa cell analyzer (BD) and BD FACSDiva 8.0.3 software were used to detect the percentage of Th1, Th2, Treg or Th17 cells.

### Detection of IFN-γ, IL-4, RORγt and Foxp3 mRNA Relative Expression

TRIzol (Invitrogen) was used to extract total RNA in lung tissues according to manufacturer’s instruction. The cDNA was synthesized with cDNA synthesis kit (K1622) (ThermoFisher) by reverse transcription according to the manufacturer’s instructions. A realtime PCR assay was then performed in 1× superreal preMix plus (SYBR Green) (FP205-02) mixed with 0.2 mM forward and reverse primers. The mRNA amounts of test genes were normalized to the amount of β-actin. The primers in the study were shown below: β-actin (FP: GAC​CCA​GAT​CAT​GTT​TGA​GAC​CT; RP: TCC​AGG​GAG​GAA​GAG​GAT​GC); RORγt (FP: CGCACCAACCTCTTTTCA CG; RP: TGG​CAA​ACT​CCA​CCA​CAT​ACT​G); Foxp3 (FP: CTTCAAGTACCACAA TATGCGACC; RP: GCG​AAC​ATG​CGA​GTA​AAC​CAA); IFN-γ (FP: CTCAAGT GGCATAGATGTGGAAG; RP: TGA​CCT​CAA​ACT​TGG​CAA​TAC​TC); IL-4 (FP: GAT​AAG​CTG​CAC​CAT​GAA​TGA​GT; RP: CCA​TTT​GCA​TGA​TGC​TCT​TTA​GG).

### Sample Collection and Metabolomics Profiling

Blood was collected with anticoagulant tubes containing anticoagulant EDTA, and then centrifuged at 4°C, 1,500 g for 15 min. All the plasma was stored at −80°C. The plasma sample was collected and detected as described in our previous study (Zhou et al., 2022).

To evaluate the sample qualification, multivariate statistical analysis such as orthogonal partial least squares discriminant analysis (OPLS-DA) were used in the study. The *R* ^2^ and Q^2^ values in the OPLS-DA model were used to assess the goodness of fit. Heatmap, cluster analysis, pathway analysis were conducted with the MetaboAnalyst web tool (https://www.metaboanalyst.ca/). Enrichment analysis was performed with the cytoscape software. Finally, we screen significantly different metabolites in plasma.

### Gut Microbiota Analysis

Total genome DNA was extracted by CTAB/SDS method, and V3-V4 regions in the 16S rDNA of samples were amplified. The following primers were used: 16S V3-V4: 341F- 806R, 18S V9: 1380F-1510R, ITS1: ITS1F- ITS2R. 16S rDNA genes were amplified using the specific primer with the barcode. PCoA (Principal Co-ordinates Analysis) was used to study the similarity or difference of sample community composition. LEfSe ((LDA Effect Size) analysis method was used for the quantitative analysis of biomarkers in the two groups.

### Statistical Analysis

All statistical analysis were performed by Prism 7.0 in this study. To analyze the difference between two groups, the *t*-test, one-way ANOVA test (Turkey or Dunnett), Wilcoxon rank-sum test were used. Data were shown as mean ± SD, and *p* < 0.05 was considered statistically significant. R software (version 3.5.1) by a hierarchical clustering algorithm and Pearson correlation analysis were used to describe the relevance between the gut microbiota, the metabolites, and the immunological index.

## Result

### OVA Sensitization Induced Eosinophilic Asthma in BALB/C Mice

**FIGURE 1:**
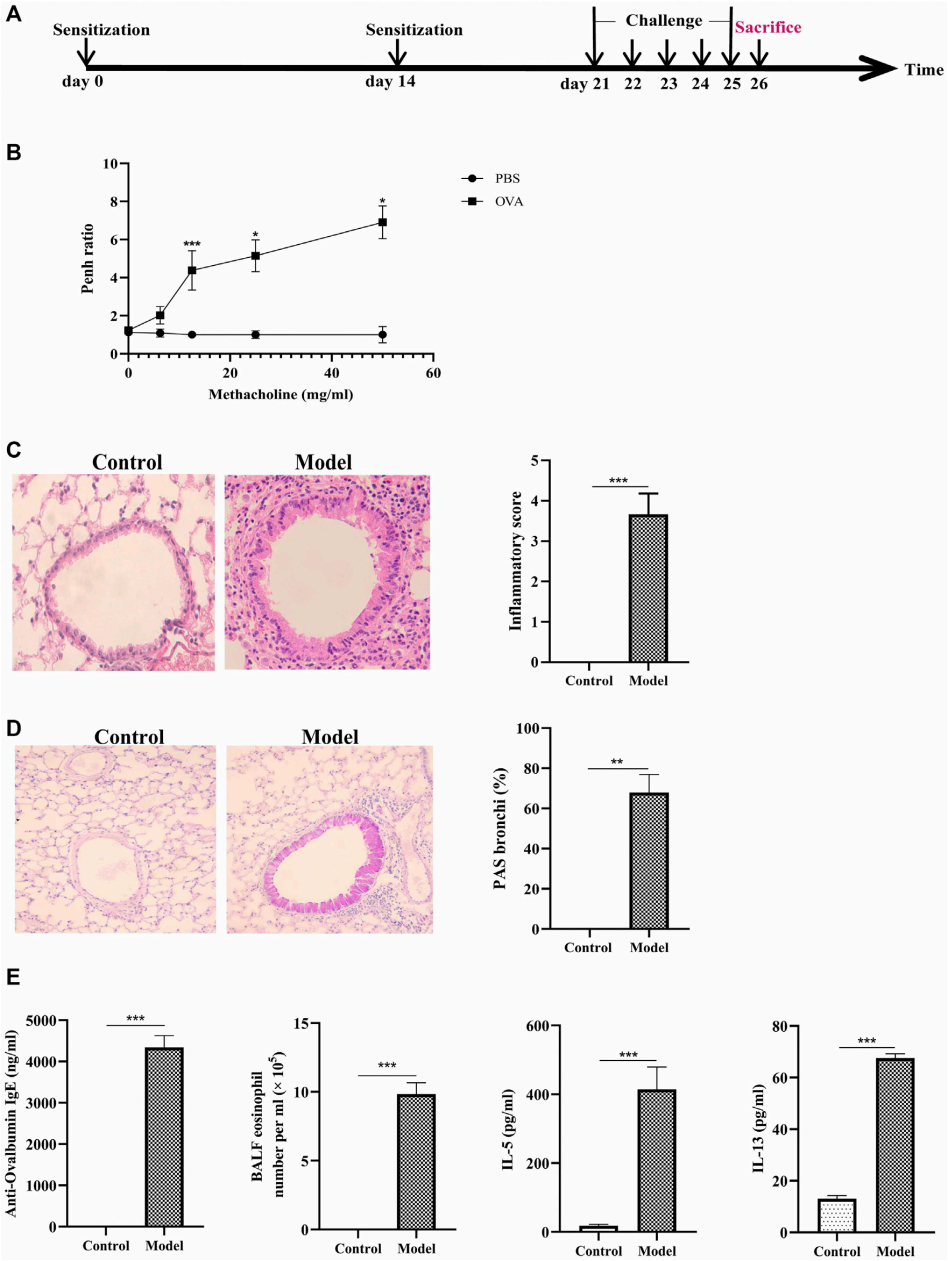
Construction of eosinophilic asthma mice model and evalution of the model. **(A)** Experimental schema for eosinophilic asthma mice mode; **(B)** AHR in response to increasing doses of MCh by monitoring penh values. (average Penh over the 5-min time interval with methacholine divided by the average Penh over the 5-min interval with PBS); **(C)** HE (Hematoxylin/eosin) staining and inflammatory score of lung tissue; **(D)** PAS staining and goblet cell hyperplasia percentage of lung tissue; **(E)** Eosinophil counts in BALF, the detection of specific OVA-IgE, IL-5, IL-13 in control and model group, BALF was collected 24 h after last challenge, *p < 0.05, **p < 0.01, ***p ≤ 0.001. All the values are expressed as mean±SEM. *n* = 4, 5 animals per group.

To induce type-2 high allergic asthma (eosinophilic asthma), we immunized BALB/c mice with OVA and aluminum adjuvant (Figure 1A). The increasing of inflammatory cells and goblet cell hyperplasia were seen in the eosinophilic asthma model group (Figures 1C,D). The airway resistance was obvious in eosinophilic asthma compared to control mice which can be shown in Figure 1B.

Next, eosinophils and the related cytokines in BALF were detected in the two groups. The cytokines of IL-4, IL-5, and IL-13 in BALF significantly increased in eosinophilic asthma mice compared to the mice in the control group (Figure 1E). Meanwhile, OVA-specific IgE in the serum of model group also increased (Figure 1E). These data suggested that the eosinophilic asthma mice model was successfully established.

### The Imbalance of Th1/2, Treg/Th17 Cells Exist in the Eosinophilic Asthma Mice Model

**FIGURE 2:**
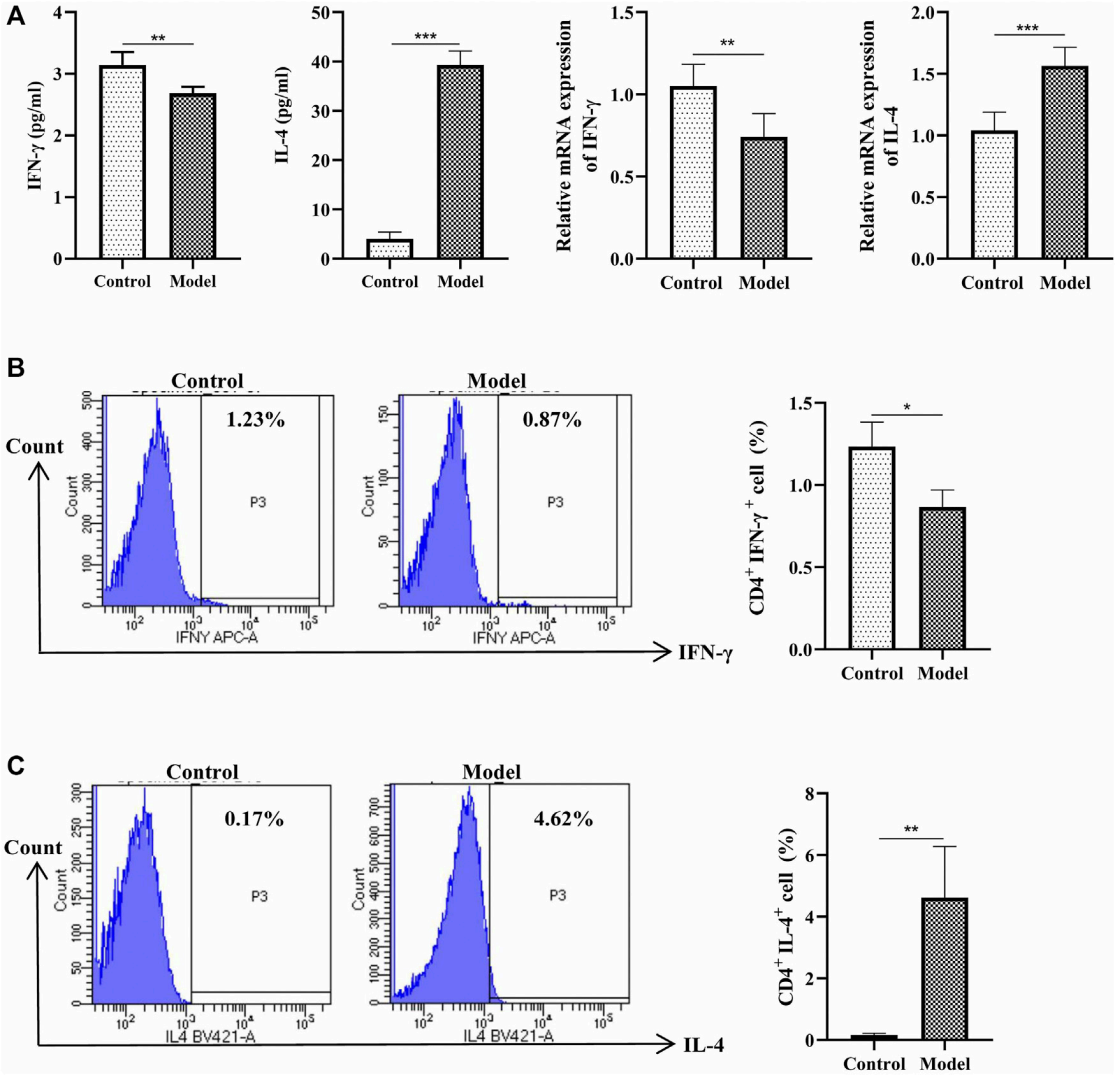
Detection of Th1 and Th2 cells. **(A)** Left:Detection of IFN-γ, IL-4 in BALF, BALF was collected 24 h after last challenge, they were detected as described in the protocol; Right: Relative expression of IFN-γ mRNA and IL-4 mRNA detected by RT-qPCR **(B)**Left: Intracellular staining of CD4 and IFN-γ in the door CD3^+^ T cells 24 h after last challenge; Right:Statistic data of Th1 cells 24 h after challenge; **(C)** Left: Intracellular staining of IL-4 and CD4 in the door CD3^+^ T cells 24 h after last challenge; Right: Statistic data of Th2 cells 24 h after challenge. *p < 0.05, **p < 0.01, ***p ≤ 0.001. All the values are expressed as mean±SEM. *n* = 5 animals per group.

The IFN-γ cytokine, the IFN-γ mRNA are downregulated, the cytokine of IL-4 and the IL-4 mRNA are upregulated (Figure 2A). Meawhile, the percentage of Th1 cells (CD3^+^CD4+IFN-γ+) are decreased, while the percentage of Th2 cells (CD3^+^CD4^+^ IL-4+) are increased (Figure 2B). These data suggested an imbalance of Th1/Th2 is exist in this eosinophilic asthma mice model.

**FIGURE 3:**
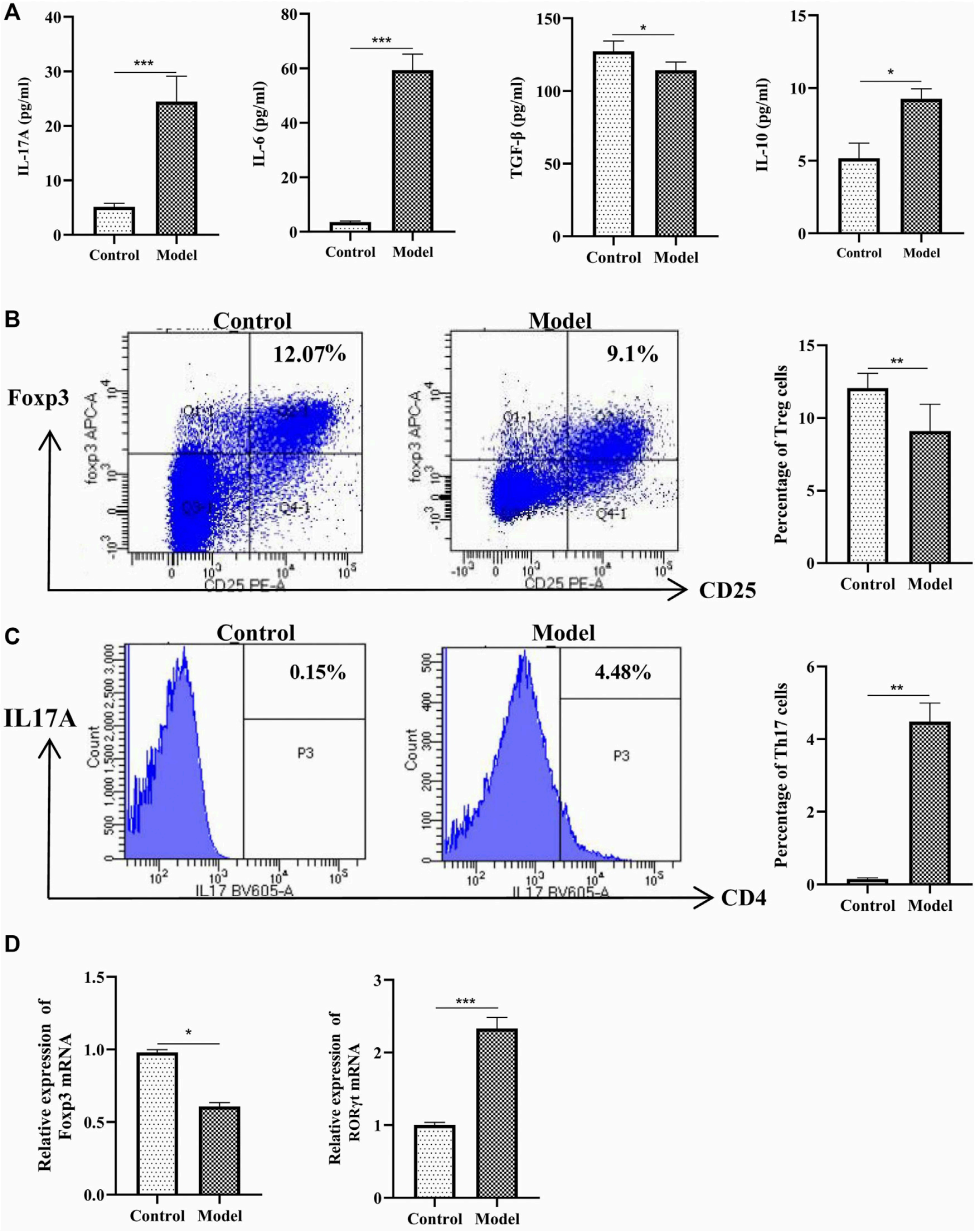
Detection of Treg and Th17 cells. **(A)** Detection of IL-17A, IL-6, TGF-β and IL-10 in BALF, BALF was collected 24 h after last challenge, they were detected as described in the protocol; **(B)**Left:Intracellular staining of CD25 and Foxp3 in the door CD4^+^ T cells 24 h after challenge; Right:Statistic data of Treg cells 24 h after challenge; **(C)** Left: Intracellular staining of IL17A and CD4 in the door CD3^+^ T cells 24 h after challenge; Right: Statistic data of Th17 cells 24 h after challenge; **(D)** Relative expression of Foxp3 mRNA and RORγt mRNA detected by RT-qPCR. *p < 0.05, **p < 0.01, ***p ≤ 0.001. All the values are expressed as mean±SEM. *n* = 4,5 animals per group.

Next, we analyzed the percentage of Treg and Th17 cells in the OVA-induced eosinophilic asthma mice model. In our study, Treg cells were decreased significantly, while Th17 cells were increased significantly (Figures 3B,C). Moreover, associated cytokines of Treg cells (TGF-β, IL-10) were significantly decreased (Figure 3A), and the Th17 cells associated with cytokines IL17 and IL-6 in BALF were increased (Figure 3A). Consistently, at the mRNA level, the Treg-related transcription factor Foxp3 was downregulated, and Th17-related transcription factor RORγt was upregulated (Figure 3D). These data suggested an imbalance of Treg/Th17 in this eosinophilic asthma mice model.

### Different Metabolites Plasma in Eosinophilic Asthma Mice Model

**FIGURE 4:**
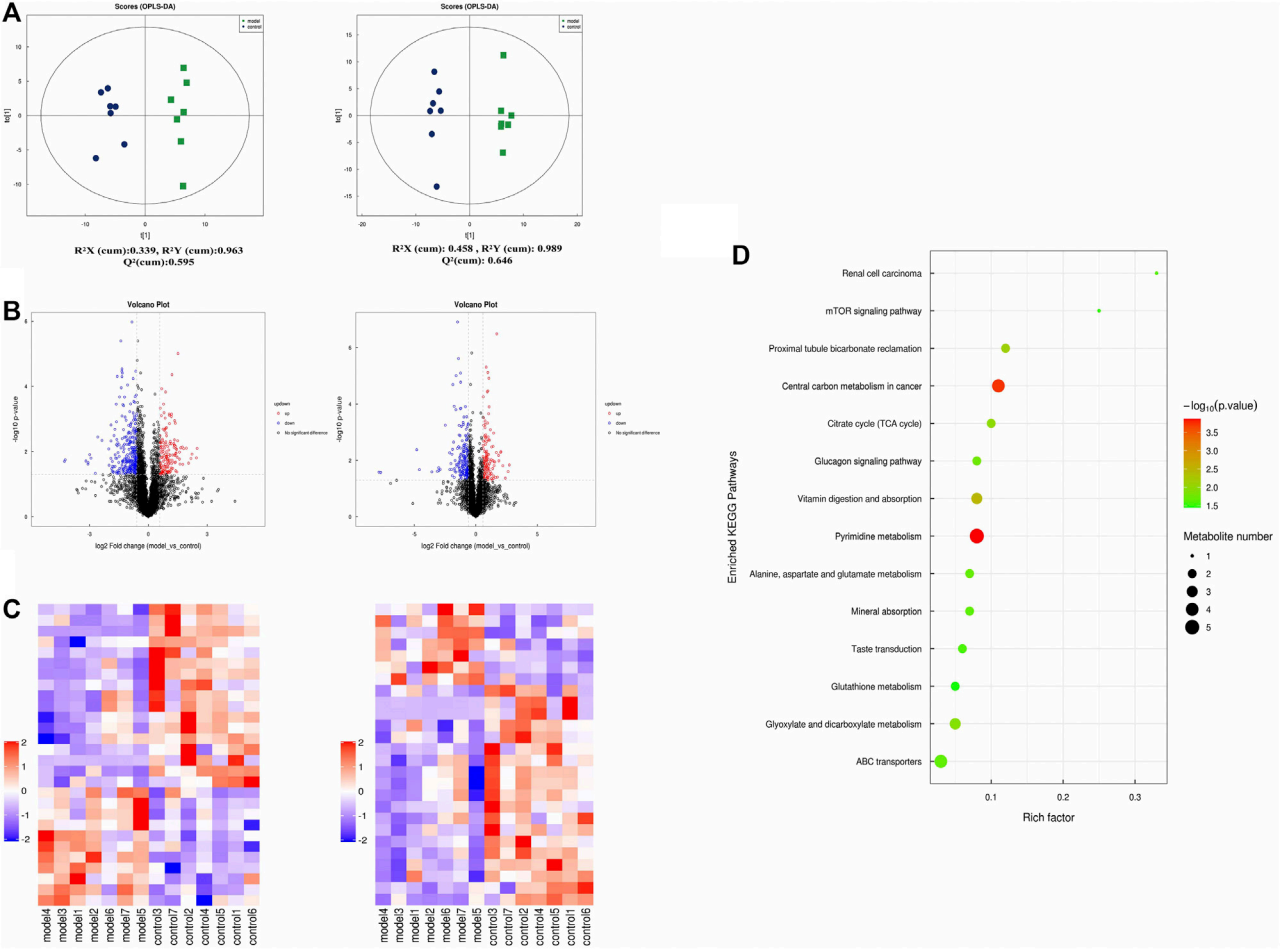
Untargeted plasma metabolome detection. **(A)** The OPLS-DA scatter plot under positive and negative ion modes; **(B)** Volcano map of different metabolites in positive and negative ion mode. (The up-regulated differential metabolites were red, FC > 1.5, *p* value <0.05; The down-regulated differential metabolites were blue, FC < 0.67, *p* value <0.05.) **(C)** The clustering results of hierarchical cluster analysis based on the significantly different metabolites between groups under positive and negative modes. **(D)** Enriched KEGG pathways based on significant different metabolites between control group (*n* = 7) and model group (*n* = 7).

To further understand the related factor of eosinophilic asthma, an untargeted metabolomics assay was performed. OPLS-DA analysis, a supervised method for pattern recognition, was performed on the data comparing the control and the model groups. As shown in Figure 4, groups in positive and negative nodes were separated in the OPLS-DA score plots (Figure 4A) with satisfactory goodness of fit. Overall, 55 different metabolites were detected; 18 metabolites were upregulated, and 37 metabolites were downregulated (Figure 4B; Table 1). The results of hierarchical cluster analysis are shown in Figure 4C; metabolites clustered in the same cluster have similar expression patterns, may have similar functions, or participate in the same metabolic process or cellular pathway.

**TABLE 1:** Differentiated plasma metabolites between model and control groups.

Metabolites	ESI	VIP	Fold Change	*p* Value
Beta-octylglucoside	+	1.131464	0.378592156	4.03E-06
Pro-hyp	+	1.559487	0.481080359	9.01E-05
1-behenoyl-2-hydroxy-sn-glycero-3-phosphocholine	+	1.20523	0.706403289	0.000112
Imidazoleacetic acid	+	1.199073	1.264756258	0.000172
1-Palmitoyllysophosphatidylcholine	+	1.64467	0.790425533	0.001203
1-lignoceroyl-2-hydroxy-sn-glycero-3-phosphocholine	+	1.478166	0.700320405	0.002041
2-deoxy-d-glucose	+	3.343785	0.852229227	0.002636
Taurohyocholic acid	+	1.420785	1.881183802	0.002657
Fraxetin	+	1.046128	0.602647196	0.003539
DL-glutamine	+	2.706991	1.236530723	0.003757
l-pyroglutamic acid	+	4.784439	1.30721999	0.00428
Captopril	+	1.09852	1.60715764	0.010905
1-heptadecanoyl-sn-glycero-3-phosphocholine	+	1.245238	0.867062765	0.011883
2-methylbutyryl-l-carnitine	+	1.097752	0.695498651	0.015584
N.omega.-hydroxy-nor-l-arginine	+	3.761146	0.58632938	0.019692
Arachidonoylthiophosphorylcholine	+	3.194667	0.73748276	0.021846
Didodecyl 3,3′-thiodipropionate oxide	+	7.401258	0.118898778	0.024455
(+)-.alpha.-tocopherol	+	2.632343	1.31377735	0.027809
1,2-dipalmitoleoyl-sn-glycero-3-phosphocholine	+	1.021394	0.71138672	0.028041
1-hexadecyl-2-(9z-octadecenoyl)-sn-glycero-3-phosphocholine	+	2.118359	1.419830373	0.029925
Didodecyl 3,3′-thiodipropionate	+	9.705313	1.177332401	0.036962
DL-Indole-3-lactic acid	+	2.483305	0.510177656	0.037305
l-Norleucine	+	1.200297	2.192440841	0.04081
N-alpha-acetyl-l-lysine	+	1.143018	2.02499295	0.042362
Erucamide	+	4.504219	0.468475768	0.042909
Androstan-3-ol-17-one 3-glucuronide	+	1.229974	0.428586777	0.043986
3-hydroxyanthranilic acid	+	1.176436	1.56196739	0.04645
Flumethasone pivalate	+	1.466925	0.446652483	0.048715
Pyridoxal	−	1.041898	0.653730059	0.000499
Myo-inositol	−	1.132384	0.718497076	0.000858
1,5-anhydro-d-sorbitol	−	1.551254	0.667099073	0.00154
Taurocholate	−	5.049303	3.614488366	0.006614
Ethylenediaminetetraacetic acid	−	30.02436	0.671296218	0.006731
Leucine	−	3.909682	1.655640658	0.009614
Malate	−	3.36886	0.466826514	0.010515
l-Ascorbic acid	−	1.220481	0.548166624	0.010813
Pseudouridine	−	2.2885	1.283592485	0.013045
Citrate	−	5.707591	0.796622739	0.013066
Propanoic acid, 3-[[[2-[(aminoiminomethyl)amino]-4-thiazolyl]methyl]thio]-	−	1.979877	0.840032629	0.013144
Dihydroxyacetone	−	2.309582	0.796052851	0.013892
Indole-3-carboxaldehyde	−	1.869297	0.55207048	0.015098
Dihydroisoferulic acid	−	1.017208	0.745015711	0.018041
l-dihydroorotate	−	2.074576	0.78453178	0.021762
2,6-di-tert-butylphenol	−	7.03295	0.126136357	0.022794
Acetylglycine	−	1.816961	0.838562471	0.023526
Valerenic acid	−	4.909679	0.114797002	0.024352
Dodecanoic acid	−	2.350182	0.630639683	0.025552
Uridine	−	1.279404	1.392006018	0.028122
(r)-2-hydroxystearic acid	−	1.521972	1.599680456	0.030486
Glutamine	−	2.540492	1.199525586	0.034038
5a,6-anhydrotetracycline	−	1.489606	0.837611418	0.037084
Oleic acid	−	20.06777	1.317683351	0.037228
His-ser	−	2.790828	0.745949054	0.041775
N-acetyl-d-glucosamine 6-phosphate	−	1.008122	0.567914407	0.047293

In addition, metabolites were grouped through pathway analysis based on the KEGG database. Interestingly, 14 different KEGG pathways showed statistical significance (Figure 4D). Interestingly pyrimidine metabolism, vitamin digestion, and absorption, glyoxylate, and dicarboxylate metabolism pathway etc., were obviously upregulated in eosinophilic asthma mice.

### Metabolites Biomarkers in Plasma Associated With Th1, Th2, Treg and Th17 Cells in Eosinophilic Asthma Mice Model

**FIGURE 5:**
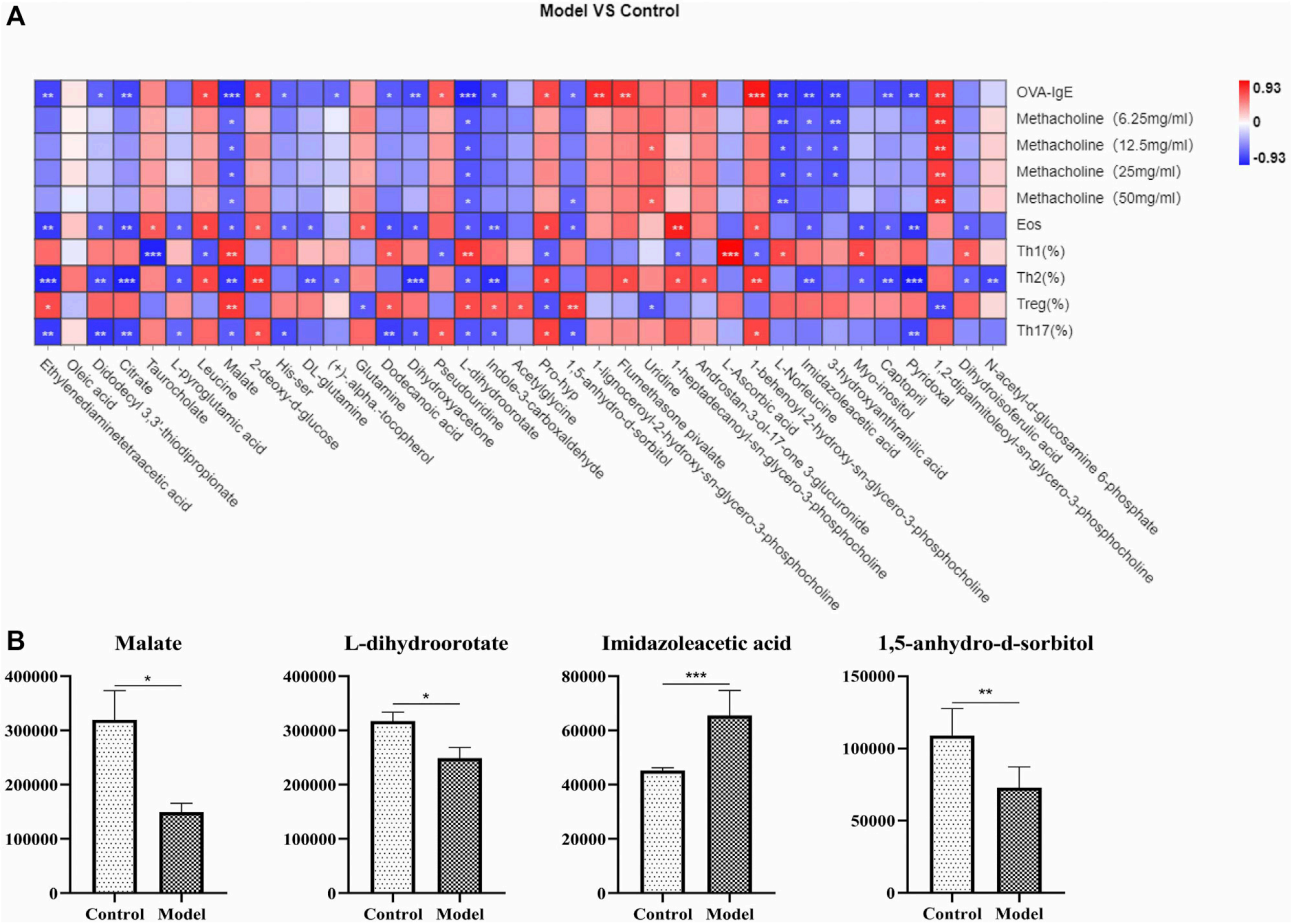
Correlation analysis of metabolic biomarkers and Th1, Th2, Treg, Th17 cells in OVA induced eosinophilic asthma model. **(A)** Correlation plot showing the relationship between the differential metabolites in plasma and the key immune indicators of eosinophillic asthma including OVA-IgE, Penh value and eosinophil number in BALF. **(B)** Biomarkers of metabolic biomarkers in eosinophilic asthma associated with Th1, Th2, Treg and Th17 cells, followed by Malate, l-dihydroorotate, Imidazoleacetic acid and 1,5-anhydro-d-sorbitol. *p < 0.05, **p < 0.01, ***p ≤ 0.001. All the values are expressed as mean±SEM. *n* = 7 animals per group.

To further understand the metabolites associated with eosinophilic asthma, spearman analysis was performed using R software. To explore plasma biomarkers associated with Th1/2, Treg/Th17 balance, differential metabolites associated with Treg cells, Th17 cells, OVA-IgE, Penh value, and eosinophil number were analyzed (Figure 5A). In this study, malate, l-dihydroorotate were associated with Th1, Th2, Treg cells, Th17 cells, OVA-IgE, Penh value (Mch: 6.25, 12.5, 25, 50 mg/ml) and eosinophil number (Eos). Interestingly, imidazoleacetic acid in plasma of eosinophilic asthma mice model was upregulated and associated with Th2 cells in with OVA-IgE, Penh value (Mch:6.25, 12.5, 25 mg/ml) and eosinophil number (Eos) on a relevant basis. While 1,5-anhydro-d-sorbitol were downregulated and associated with Treg and Th17 cells based on related with OVA-IgE, Penh value (Mch: 50 mg/ml) and eosinophil number (Eos) (Figure 5B).

### Variation of Gut Microbiota and the Biomarkers of Gut Microbiota in Response to OVA-Induced Eosinophilic Asthma Modeling

**FIGURE 6:**
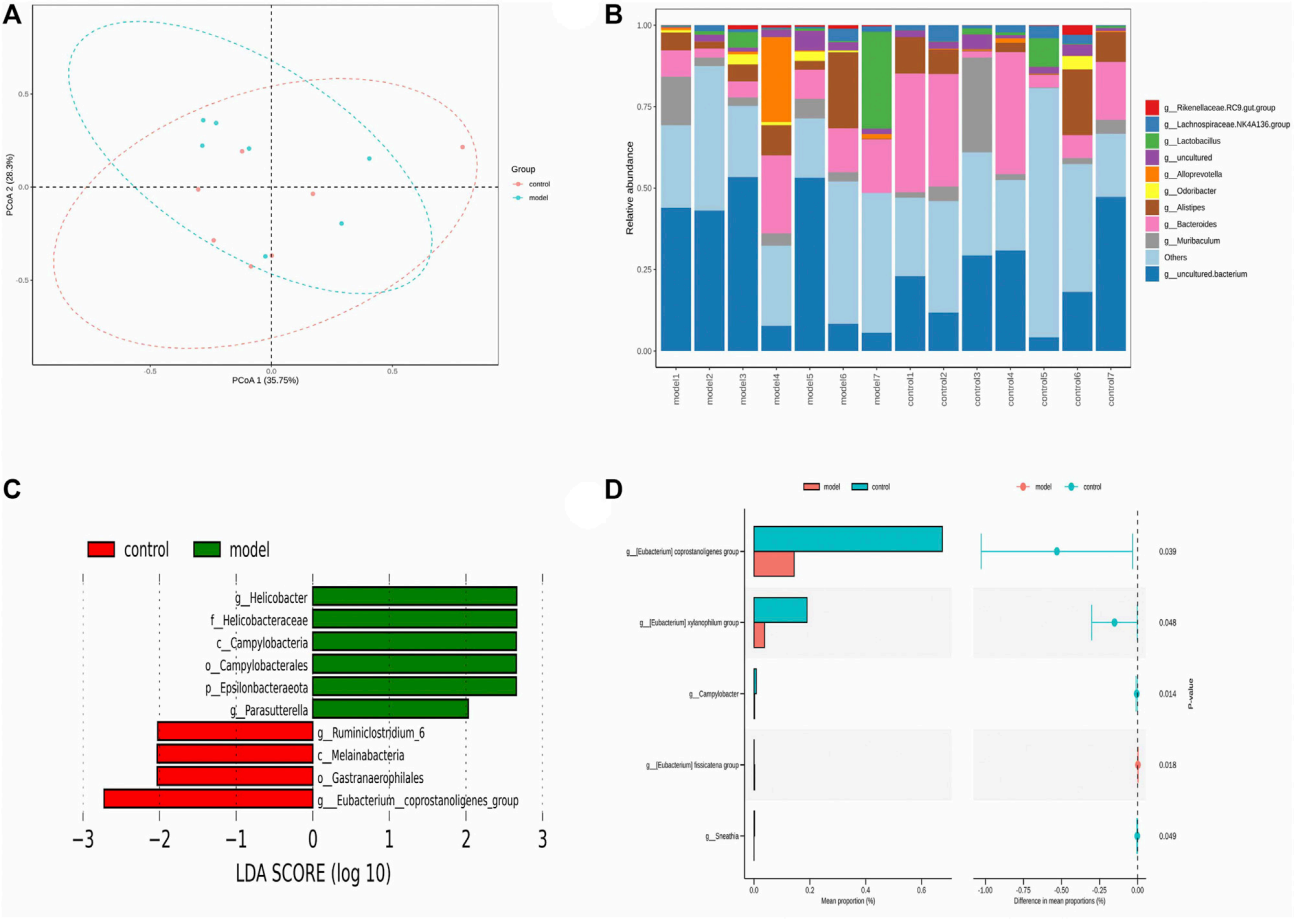
Gut microbiota biomarker in OVA induced eosinophilic asthma BALB/c mice model. **(A)** Principal Co-ordinates Analysis (PCoA) of control group (*n* = 7) and model group (*n* = 7). **(B)** Community structure composition map of gut microbiota. **(C)** LEfSe (LDA Effect Size) analysis of different species in control group and model group (LDA > 2). **(D)** STAMP analysis of different gut microbiota (*p* < 0.05). *n* = 7 animals per group.

The pathogenesis of asthma is complex. To analyze this process, we profiled the overall variation of gut microbiota to determine whether OVA-induced eosinophilic asthma mice modeling may impact the microbial populations. As shown in Figure 6 A and Figure 6 B, the structure of the gut microbiota was changed. To explore potential biomarkers of eosinophilic asthma, microbial phylotypes in response to OVA-induced eosinophilic asthma mice modeling were identified with LEfSe. Specifically differentiated phylotypes between model and control group were identified: *g_Ruminiclostridium 6*, *c_Melainabacteria*, *o_Gastranaerophilales*, *g_Eubacterium_coprostannoligenes_group* in the control group; genus *Helicobacter*, family Helicobacteraceae, class order *Campylobacterales*, phylum *Epsilonbacteraeota*, genus *Parasutterella* were important microbiota in the eosinophilic asthma model group (Figure 6C).

To further investigate the gut microbiota biomarkers, the STAMP difference analysis method was used to analyze the abundance of species in two groups. Different abundance was found in the Genus *Coprostanoligenes*, genus *Xylanophilum*, genus *Geodermatophilus*, genus *Campulobacter*, genus *fissicatena* group, and genus *Sneathia* (*p* < 0.05) (Figure 6D).

### Variation of Gut Microbiota and Key Phylotypes of Gut Microbiota, Differential Plasma Metabolites Associated With Th1/2 and Treg/Th17

**FIGURE 7:**
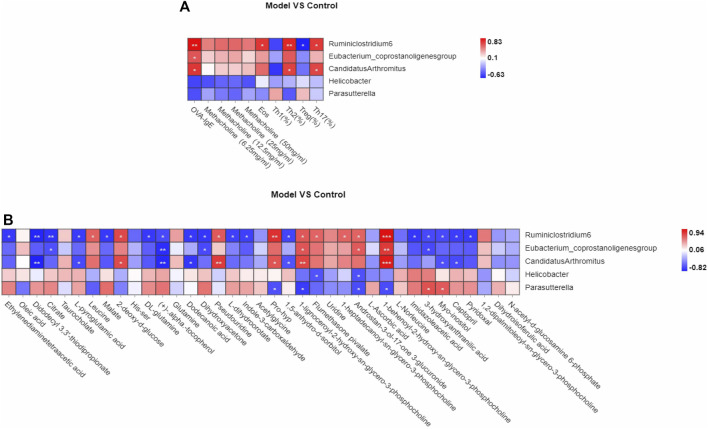
Correlation analysis of Th1, Th2, Treg, Th17 cells, differential metabolites biomarker respectively with gut microbiota biomarkers in OVA induced eosinophilic asthma model. **(A)** Correlation analysis between gut microbiota biomarker and the key immune indicators of eosinophilic asthma including OVA-IgE, Penh value and eosinophil number in BALF, Th1, Th2, Treg and Th17 cells **(B)** Biomarkers of metabolic biomarkers associated with gut microbiota biomarkers in eosinophilic asthma mice model. *p < 0.05, **p < 0.01, ***p ≤ 0.001. *n* = 7 animals per group.

The pathogenesis of asthma is complex. To analyze this process, we profiled the overall variation of gut microbiota and plasma metabolites to determine whether OVA-induced eosinophilic asthma mice modeling may impact the immune balance of Th1/Th2 and Treg/Th17. Pearson associated analysis was used to further investigate the relationship between gut microbiota and eosinophilic asthma, gut microbiota and differential plasma metabolites. As shown in Figure 7 A, genus *Ruminiclostridium 6* and genus *Candidatus Arthromitus* were associated with OVA-IgE, Eos, Th2 cells, Treg cells and Th17 cells. Interestingly, genus *Candidatus Arthromitus* is associated with OVA-IgE, Th2 cells and Th17 cells (Figure 7A). However, the differential gut microbiota biomarker are all irrelevant with penh value which was applied to calculate the AHR.

Interestingly, the plasma metabolite biomarkers of malate, l-dihydroorotate are associated with genus *Ruminiclostridium 6*, they were all associated with Th1/2 and Treg/Th17 cell balance, while imidazoleacetic acid is associated with genus *Ruminiclostridium 6* which is associated with Th1/2 balance. Among the differential plasma metabolites, 1,5-anhydro-d-sorbitol is associated with genus *Ruminiclostridium 6* and genus *Candidatus Arthromitus.*

## Discussion

Treg cells play an essential role in allergic asthma by suppressing T helper effector cells such as Th1, Th2, and Th17 cells; which can inhibit inflammatory cell infiltration in lung tissues and induce IgE switching to IgG4 (Palomares et al., 2010; Rauber et al., 2019). Our study highlights the immune balance of Th1/2 and Treg/Th17 cells in eosinophilic asthma mice model.

In our study, we successfully constructed a eosinophilic asthma mice model predominantly infiltrated by eosinophils. The ovalbumin (OVA) sensitization model can mimic the prominent eosinophilic response of severe asthma in humans (Holgate and Polosa, 2006). The spleen is the largest secondary lymphoid organ in the body and T cells in the spleen organ are the key effectors of the adaptive immune system (Lewis et al., 2019). Therefore, we detected the Treg and Th17 cells in the spleen tissue to evaluate the immune balance of Treg/Th17 cells in the eosinophilic asthma mice model. Treg cells were decreased while Th17 cells were increased all obviously in the eosinophilic mice model group. To further confirm the imbalance of Treg/Th17, we also detected TGF-β, IL10, IL17A and IL-6 cytokines in BALF. TGF-β plays an important role in the maintenance and induction of Treg cells, while IL-10 is produced mainly by many immune cells such as monocytes, Treg, Breg cells, natural killer (NK) cells, macrophages, dendritic cells (DCs), and innate lymphoid cells (ILCs). We found that TGF-β and IL-10 were significantly decreased in the model group, which is consistent with other studies (Tagkareli et al., 2021). In clinical, IL-17A mRNA and IL-17A protein are all increased in asthmatic patients compared with healthy subjects. In this study, IL17A and IL-6 were upregulated in the eosinophilic asthma model group, which is consistent with the clinical results (Laan et al., 2002). Moreover, Foxp3 and RORγt as the transcription factor of Treg cells and Th17 cells were all detected. The Foxp3 mRNA expression was downregulated, while the RORγt mRNA expression was upregulated. The imbalance of Treg/Th17 cells existed in the eosinophilic asthma mice model, the imbalance of Th1/2 is also exist in the mice model of our study.

To explore new biomarkers of the eosinophilic asthma mice model, we used untargeted plasma metabolomics and 16S rDNA sequencing technology to examine the differential metabolites and microbiota between the eosinophilic asthma mice model and control group. A previous study found that 12/15-Lipoxygenase can regulate IL-33-induced eosinophilic asthma in mice (Miyata et al., 2021). In our study, 15 significant metabolism pathways associated with eosinophilic asthma were identified. Among them, 4 metabolites such as malate, l-dihydroorotate, imidazoleacetic acid and 1,5-anhydro-d-sorbitol were all associated with Treg/Th17 cell balance in eosinophilic asthma, while malate, l-dihydroorotate, imidazoleacetic acid were associated with Th1/2 cell balance in eosinophilic asthma. Malate is participate in the tricarboxylic acid (TCA), which is important in the synthesis of aspartate to maintain proliferation of the Th cells. It can combine with aspartate to regulate the expression of genes associated with T cell activation (Bailis et al., 2019). While, there are no reports about the specific role of malate in allergic asthma disease, it may participate in the T cells differentiation of Th cells which is the essential immune mechanism of allergic asthma disease. Our data reported for the first time that malate, l-dihydroorotate, imidazoleacetic acid and 1,5-anhydro-d-sorbitol can act as biomarkers in eosinophilic asthma disease.

The “hygiene hypothesis” is the first that proposed a link between microbes and allergy disease (Stiemsma and Turvey, 2017). So far, more and more evidence has shown that there is an association between bacterial components and asthma. Studies have shown that the imbalance of “gut-lung axis” exist in asthma. Gut microbiota is linked to allergic asthma through regulating the immune response (Penders et al., 2007). In this study, genus *Ruminiclostridium 6*, genus *Candidatus Arthromitu*s were associated with the immune balance of Th1/2 and Treg/Th17 in eosinophilic asthma, while genus *Candidatus Arthromitu*s (also designated as Candidatus Savagella) is a type of segmented filamentous bacteria (SFB) can influence the immune response of the intestinal tract to resistance against some infectious diseases and it also can induce antigen-specific Th17 cells (Hedblom et al., 2018). To our surprise, genus *Ruminiclostridium 6*, genus *Candidatus Arthromitu*s were all irrelevant with penh value which is the indicator of reactive airway hyperresponsiveness.

Interestingly, malate, l-dihydroorotate, imidazoleacetic acid and 1,5-anhydro-d-sorbitol are all associated with genus *Ruminiclostridium 6.* While 1,5-anhydro-d-sorbitol is associated with genus *Ruminiclostridium 6* and genus *Candidatus Arthromitu*s. Until now, there are no related reports about them yet, while it will be a new research new research directions and new discoveries, we should combine experiments such as clinical trials and microbiota transplantation to verify our results because the gut microbiota of animals and humans is different. Besides that, more experiments are needed to confirm that these can act as biomarkers in eosinophilic asthma, they can be used as a new strategy for studying the mechanism and treatment foundation of eosinophilic asthma.

## Data Availability

The original contributions presented in the study are publicly available. This data can be found here: NCBI, PRJNA825171.
